# Effectiveness of an Educational Video in Maternity Wards to Prevent Self-Reported Shaking and Smothering during the First Week of Age: A Cluster Randomized Controlled Trial

**DOI:** 10.1007/s11121-020-01145-z

**Published:** 2020-07-22

**Authors:** Takeo Fujiwara, Aya Isumi, Makiko Sampei, Yusuke Miyazaki, Fujiko Yamada, Hisashi Noma, Kazuhide Ogita, Nobuaki Mitsuda

**Affiliations:** 1 Department of Global Health Promotion, Tokyo Medical and Dental University, 1-5-45, Yushima, Bunkyo-ku, Tokyo, 113-8519 Japan; 2 Department of Social Medicine, National Research Institute for Child Health and Development, Tokyo, Japan; 3 Department of Systems and Control Engineering, Tokyo Institute of Technology, Tokyo, Japan; 4 Department of Data Science, The Institute of Statistical Mathematics, Tokyo, Japan; 5 Department of Obstetrics and Gynecology, Rinku General Medical Center, Osaka, Japan; 6 Department of Obstetrics, Osaka Medical Center and Research Institute for Maternal and Child Health, Osaka, Japan

**Keywords:** Shaken baby syndrome, Abusive head trauma, Prevention, Infant crying

## Abstract

This study aimed to investigate whether watching an educational video on infant crying and the dangers of shaking and smothering within 1 week after delivery at maternity wards reduces self-reported shaking and smothering, at a 1-month health checkup. A cluster randomized controlled trial, stratified by area and hospital function, was employed in 45 obstetrics hospitals/clinics in Osaka Prefecture, Japan. In the intervention group, mothers watched an educational video on infant crying and the dangers of shaking and smothering an infant, within 1 week of age, during hospitalization at maternity wards, without blinding on group allocation. Control group received usual care. A total of 4722 (*N* = 2350 and 2372 for intervention and control group, respectively) mothers who delivered their babies (still birth and gestational age < 22 weeks were excluded) between October 1, 2014, and January 31 were recruited. Outcomes were self-reported shaking and smothering behaviors, knowledge on infant crying and shaking, and behaviors to cope with infant crying, assessed via a questionnaire at a 1-month health checkup. In all, 2718 (*N* = 1078 and 1640) responded to the questionnaire (response rate: 58.3%), and analytic sample size was 2655 (*N* = 1058 and 1597 for intervention and control group, respectively). Multilevel analysis was used to adjust for correlation within the cluster. Prevalence of shaking was significantly lower in the intervention group (0.19%) than in the control group (1.69%). Intention-to-treat analysis showed an 89% reduction in the reported prevalence of self-reported shaking (OR: 0.11, 95% CI: 0.02–0.53) due to watching the educational video. However, self-reported smothering behavior showed no significant reduction (OR: 0.66, 95% CI: 0.27–1.60). No side effects were reported. Watching an educational video on infant crying and the dangers of shaking and smothering within 1 week after delivery at maternity wards reduced self-reported shaking at 1 month of age. UMIN Clinical Trial Registry UMIN000015558.

**Electronic supplementary material:**

The online version of this article (10.1007/s11121-020-01145-z) contains supplementary material, which is available to authorized users.

Shaking of infants can lead to abusive head trauma (AHT), or shaken baby syndrome (SBS), and considered as a form of lethal child abuse (Duhaime et al. 1998). The proportion of deaths among infants with AHT is 10% in Japan and up to 23% in Western countries (Hobbs et al. 2005; Keenan et al. 2003; Fujiwara et al. 2008; Fanconi and Lips 2010; Talvik et al. 2006), and even if the infants survived, two-thirds suffered from the devastating effects of brain damage, including blindness, paralysis, behavior problems, or learning disabilities (Sieswerda-Hoogendoorn et al. 2012).

Another fatal form of infant abuse is smothering, which can result in death due to suffocation (Southall et al. 1987; Hwa et al. 2015; Meadow 1989). Approximately 2–3% of young children have experienced shaking and smothering worldwide (Runyan et al. 2009; Yamada and Fujiwara 2014). Hence, effective interventions are needed to prevent and raise awareness of the dangers of these behaviors.

## Previous Studies to Prevent Shaking and Smothering

Shaking and smothering behaviors are known to be triggered by prolonged infant crying (Barr et al. 2006; Fujiwara et al. 2016b), and educational material on infant crying can have a preventive effect when provided appropriately. For example, Dias et al. showed that educating mothers on the dangers of shaking in a hospital-based study reduced 51% of AHT cases admitted to hospitals (Dias et al. 2005). Barr et al. developed educational materials on infant crying which promoted walk-away behavior as a coping mechanism for parents frustrated by prolonged crying, in a population-based study delivered through the maternity ward and home visits by public health nurses (Barr et al. 2009a). The researchers found that the intervention was effective in increasing walk-away behavior, but the actual reduction of admitted AHT cases among infants was not confirmed in North Carolina, the United States (Zolotor et al. 2015), and British Columbia, Canada (Barr et al. 2018). In addition, parent education in the hospital by maternity nurses on how to cope with infant crying found significant reduction of shaking injury among infants (Altman et al. 2011). Therefore, using educational materials in maternity wards might be an efficient and effective way to save cost and time.

To the best of our knowledge, however, no previous studies have investigated the impact of educational material, shown at the maternity ward to mothers just before they were discharged with their infant, on the prevention of both shaking and smothering. It has been argued that providing information on infant crying right after delivery might be too early for the exhausted mother considering the peak of crying occurs only at 6 weeks (Hunziker and Barr 1986). On the contrary, some have stated that the period immediately after delivery is a good opportunity to disseminate important information on infant crying and the dangers of shaking and smothering, similar to providing information on child safety seats (Liu et al. 2016).

Further, previous randomized controlled trials of educational materials to prevent SBS have shown that such materials were effective in increasing knowledge on crying and behaviors to deal with the crying (Barr et al. 2009a). However, no trials have shown the effectiveness of such interventions to decrease shaking and smothering behaviors per se. Previous studies showing the effectiveness of educational material to prevent SBS used difference-in-difference analysis, that is, comparing between areas delivering the intervention and comparison areas in terms of the difference of incidence of AHT before and after the intervention implemented (Zolotor et al. 2015; Barr et al. 2018). As there could be a number of confounding factors in studies that do not use random allocation, such as the economic recession in 2008, the true effectiveness of the interventions remains unclear. Thus, a randomized controlled trial on the effectiveness of educational material on infant crying to prevent shaking and smothering by showing an actual decrease in these behaviors is warranted. To address this knowledge gap, the current study aimed to examine the effectiveness of an educational video in maternity wards to prevent shaking and smothering.

## The Current Study

Japan has a long-stay model of perinatal care, under which mothers are to stay at the obstetric hospital for at least 5 days after delivery, which is much longer than other developed countries. Further, new mothers would have to visit the same obstetric hospital to receive a 1-month health checkup for themselves and their baby. This system provides us with a unique opportunity to examine whether showing an educational video on infant crying during admission, i.e., at maternity wards within 1 week of delivery, is effective to prevent shaking.

Further, as the prevention of smothering behavior remains unexplored, and is not included in key interventions such as the “Period of PURPLE Crying” (R. G. Barr and National Center on Shaken Baby Syndrome 2004), there is also a need to assess the effectiveness of an educational video to prevent smothering. Therefore, in collaboration with the Ministry of Health, Labour and Welfare in Japan, we developed an educational video titled “Baby Doesn’t Stop Crying” (accessible via https://www.youtube.com/watch?v=T09gzgGUOnY&feature=c4-overview&list=UUVgZUHlkoN51FOwoNMBGjfw). The video, based on materials from the “Period of PURPLE Crying” by the National Center on Shaken Baby Syndrome (Barr et al. 2009a), explains patterns of infant crying in healthy infants, including “peak crying,” which occurs during the second month followed by a decline. A computer graphic simulation of the infant brain and an anatomical doll are used to show the devastating impact that occurs inside the infant skull when a baby is shaken, including the velocity of the skull and brain during shaking. Further, in the video, we recommended several ways, based on the coping strategies of the “Period of PURPLE Crying” (Barr and National Center on Shaken Baby Syndrome 2004), to soothe infants, such as holding (Hunziker and Barr 1986), feeding, swaddling (van Sleuwen et al. 2006), the use of rhythmic stimulation (Barr and Fujiwara 2011), or taking a break, such as stepping out of the room and away from the crying infant. The video also emphasized the importance of never shaking or smothering an infant.

In this study, we utilized a randomized trial design in maternity wards, where most of the mothers deliver, to investigate the efficacy of a video intervention on infant crying to reduce parent shaking and smothering at 1 month of age, when most of them receive health checkup. Thus, the purpose of this study is to investigate whether watching an educational video on infant crying and the dangers of shaking and smothering, within 1 week after delivery at a maternity ward, reduces self-reported shaking and smothering behaviors at 1 month of age.

## Methods

### Study Population and Protocol

A cluster randomized controlled trial was performed in participating hospitals with maternity wards in Osaka Prefecture, Japan, in 2015. Details of the protocol can be found in Supplemental Material. Located in the western part of Japan, Osaka Prefecture is Japan’s second largest prefecture with a population of around 8 million people. To account for regional variations within Osaka Prefecture and the functions of the hospitals, participating hospitals were stratified by area (seven regions based on administrative classification) and hospital functions (primary or secondary/tertiary hospital). Of the 150 hospitals with maternity wards in Osaka Prefecture, which are representative hospitals for Osaka prefecture but not for Japan, 45 agreed to participate in the study, and intervention or control groups were assigned randomly by our staff who were blinded on group allocation using stratified block randomization (block size of 2), with allocation ratio of 1:1, determined by a computer-generated randomization sequence.

Participants were mothers who delivered their baby between October 1, 2014, and January 31, 2015. Mothers who delivered still birth or delivered at < 22 weeks gestational age were excluded. In the intervention groups, participants viewed the educational video during hospitalization. Therefore, hospital staffs and participants were not blinded. Maternal knowledge and behaviors relating to crying were assessed by a questionnaire at the 1-month health checkup conducted at the same hospital. Obstetricians or nurses, who were not blinded to random allocation, distributed questionnaires to mothers at discharge, and mothers, who were not blinded either, returned the completed questionnaire to the hospital at the 1-month health checkup. The questionnaire evaluated maternal crying and shaking knowledge, and shaking and smothering behavior in the past month in response to crying and unsoothable crying, and other demographics and caregiving-related behaviors. These knowledge scales were based on previous studies (Barr et al. 2009a, b; Fujiwara et al. 2012), and the same questionnaire on shaking or smothering behaviors was used before (Yamada and Fujiwara 2014; Fujiwara et al. 2016b). Outcome measures were defined prior to analysis. The questionnaire was anonymous and would be able to be completed within 10 min. To address ethical concerns, participants in the control group viewed the educational video after submitting the questionnaire at the 1-month health checkup.

Ethics approval was granted by the Ethics Committee at the National Center for Child Health and Development (No. 785).

### Intervention Materials

The educational video was developed based on existing material, the “Period of PURPLE Crying” by the National Center on Shaken Baby Syndrome (Barr et al. 2009a). The video explains patterns of infant crying in healthy infants, including “peak crying,” which occurs during the second month and then declines and emphasizes the danger of shaking by using a computer graphic simulation of the infant brain and an anatomical doll showing the devastating impact that occurs inside the infant skull when a baby is shaken, including the velocity of the skull and brain during shaking. Hospitals were asked to show the educational video in maternity wards to mothers after delivery and when they were admitted to the hospital.

### Outcomes

Our primary outcome measures were self-reported shaking and smothering. That is, we assessed the frequency of shaking behavior in the past month using the following statement: “When your child is crying and making a scene, how many times have you violently shaken your child?”. We used the Japanese term for “violently shaking” instead of “shaking” in the questionnaire because the Japanese term “shaking” can be misinterpreted as “rocking.” Similarly, frequency of smothering was assessed with the following question: “How many times have you covered the mouth of your baby with your hands, a cushion, etc., when he/she was crying?”. The respondents selected their answer for each of these questions from the following response items during the past month: “0 times,” “1 or 2 times,” “3–5 times,” “6–10 times,” and “11 or more times.” In our analysis, these responses were dichotomized as 1 for 1 time or more and 0 for 0 times, due to low prevalence of “3–5 times,” “6–10 times,” and “11 or more times,” and following previous studies which dichotomized the same question responses (Fujiwara et al. 2016a, b; Yamada and Fujiwara 2014). Further, six secondary outcomes were assessed: crying knowledge, shaking knowledge, sharing information on crying with at least one other family member (the peak of crying, walk-away behavior, or the danger of shaking), walking away, active coping, and self-talk in response to unsoothable crying. In line with previous studies (Barr et al. 2009a, b), we transformed the behavior scales for crying and shaking knowledge, active coping, and self-talk behaviors to the range of 0–100 points, where higher scores indicated better knowledge or expected behaviors. Sharing crying information and walk-away behaviors were dichotomized, whether the behaviors occurred 0 times or 1 or more time.

### Statistical Analysis

For primary outcomes, that is, shaking and smothering, we determined that 1544–3328 participants were needed to achieve 80% power to detect an effect size of 0.5–0.7 between participants in the intervention and control groups; thus we sought to enroll about 3000 mothers.

The analytic strategy was determined in advance and independent of any results from the study. We employed intention-to-treat analysis. We did not use data for analysis if respondents were the fathers or missing for the information on their sex, did not respond to the primary outcomes, and reported that infants stayed at the neonatal intensive care unit (NICU) at the time of their 1-month checkup. Covariates which can be associated with both the intervention and outcomes based on literature, that is, area, child age, feeding, maternal education, and income (Fujiwara et al. 2016a, b; Yamada and Fujiwara 2014), were adjusted in addition to the crude model.

For continuous measurements, the mean difference between participants in the intervention group and the control was estimated using a multilevel regression model with a restricted maximum likelihood method that takes into account clustering of the hospital. For dichotomized outcomes, we estimated the odds ratio (OR) using a multilevel logistic regression model. Following the previous randomized controlled trials (Barr et al. 2009a, b), we used tests of statistical interaction for our primary outcomes to examine subgroups based on education (high school or less vs. some college or more), birth order (first child vs. subsequent child), and whether the infant cried unsoothably. All analyses were conducted using Stata SE version 14 (StataCorp, College Station, TX, 2015) in 2018. All tests were two-sided with a significance level of *p* < 0.05.

## Results

### Characteristics of Intervention and Control Groups

**Fig. 1:**
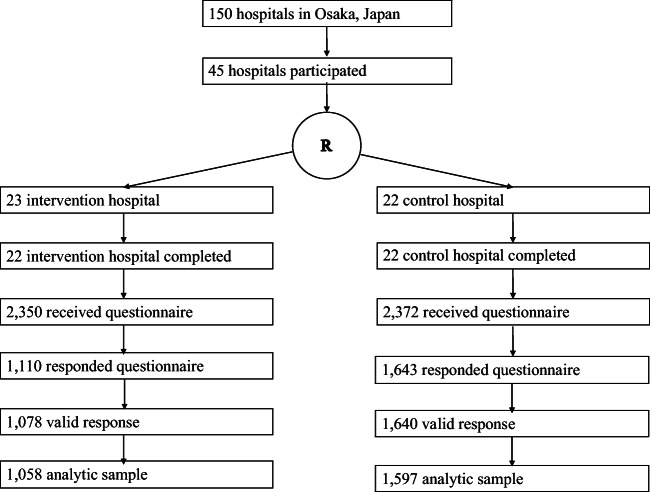
Consort diagram of this study

After randomization, 1 hospital withdrew from the study; thus in the final analyses 22 hospitals were in the intervention, and 22 in the control were included (Fig. 1). Questionnaires were distributed to 2350 and 2372 mothers from the intervention and control arms, respectively, and 1110 and 1643 participants submitted questionnaires from these arms, respectively. The response rates were as follows: overall (58.3%), intervention (47.2%), and control (69.3%). Among them, 1078 and 1640 were valid response, respectively. Of these, 63 were not used for analysis for not meeting the study criteria: 23 were the fathers or missing for sex of respondents, 26 participants missing on shaking and smothering behaviors, and 14 participants had infants who were still in the NICU (analytic sample size = 2655, intervention group *N* = 1058, and control group *N* = 1597). Although most of the demographic variables of the participants were similar in both groups, significant differences between the intervention and control group were found in area, age of infant, feeding, education, and income (Table 1). For example, 77.1% of infants were 4–5 weeks old in the control group compared with 68.8% in the intervention group, exclusive breastfeeding was higher in the control group than in the intervention group (48.1 vs. 41.7%), and maternal education with college or more was higher in the intervention group than in the control group (35.4 vs. 30.3%). Any specific harms or unintended effects in each group were reported.

**Table 1:** Demographics of sample

		Control group (*N* = 1597)		Intervention group (*N* = 1058)	
		N	%	N	%
Area	Hokusetsu	347	21.7	358	33.8
	Osaka city	382	23.9	302	28.5
	Kita Kawachi	83	5.2	23	2.2
	Naka Kawachi	46	2.9	27	2.6
	Minami Kawachi	251	15.7	57	5.4
	Senhoku	156	9.8	175	16.5
	Sennan	332	20.8	116	11.0
Age of mother	< 20 y	25	1.6	12	1.1
	20–24 y	136	8.5	86	8.1
	25–29 y	408	25.6	229	21.6
	30–34 y	555	34.8	375	35.4
	35–39 y	375	23.5	276	26.1
	40 + y	98	6.1	79	7.5
	Missing	0	0.0	1	0.1
Sex of infant	Male	790	49.5	512	48.4
	Female	806	50.5	545	51.5
	Missing	1	0.1	1	0.1
Age of infant	3 weeks or less	147	9.2	204	19.3
	4–5 w	1231	77.1	728	68.8
	6w+	183	11.5	71	6.7
	Missing	36	2.3	55	5.2
Birth order	First	846	53.0	513	48.5
	Subsequent	750	47.0	544	51.4
	Missing	1	0.1	1	0.1
Marital status	Married	1548	96.9	1029	97.3
	Unmarried or divorced	41	2.6	25	2.4
	Missing	8	0.5	4	0.4
Living with grandparents	Yes	153	9.6	119	11.3
	No	1444	90.4	939	88.8
Birth weight	< 2500 g	132	8.3	92	8.7
	2500 g+	1462	91.6	964	91.1
	Missing	3	0.2	2	0.2
Feeding	Breastfeeding only	768	48.1	441	41.7
	Mixed	769	48.2	567	53.6
	Milk only	54	3.4	40	3.8
	Missing	6	0.4	10	1.0
Unsoothable crying	Yes	480	30.1	291	27.5
	No	1089	68.2	750	70.9
	Missing	28	1.8	17	1.6
Education	High school or less	449	28.1	295	27.9
	Some college	643	40.3	380	35.9
	College or more	484	30.3	375	35.4
	Missing	21	1.3	8	0.8
Income	< 2 million yen*	56	3.5	33	3.1
	2–10 million yen	1338	83.8	857	81.0
	10+ million yen	53	3.3	73	6.9
	Missing	150	9.4	95	9.0
Watch DVD	0	1307	81.8	96	9.1
	1 time	226	14.2	855	80.8
	2+ times	10	0.6	81	7.7
	Missing	54	3.4	26	2.5

100 yen is equivalent to USD1

### Main Intervention Effects

Table 2 shows the results of primary outcome measures (i.e., shaking and smothering). Prevalence of shaking was significantly lower in the intervention group (0.19%) than in the control group (1.69%). After adjusting for area, infant age, feeding, education, and income, the intervention arm showed lower odds on self-reported shaking behaviors (OR: 0.12, 95% CI: 0.03–0.54). On the other hand, the intervention did not significantly show the difference on smothering behaviors (in crude model, OR: 0.58, 95% CI: 0.23–1.45). No side effects were reported.

**Table 2:** Comparison of shaking and smothering behaviors by intervention and control groups

		Prevalence	Crude		Adjusted*	
		*n* (%)	OR	95% CI	OR	95% CI
Shaking	Control	27 (1.69)	Ref		Ref	
	Intervention	2 (0.19)	0.11	0.02–0.53	0.12	0.03–0.54
Smothering	Control	16 (1.00)	Ref		Ref	
	Intervention	7 (0.66)	0.66	0.27–1.61	0.58	0.23–1.45

*Adjusted for covariates (i.e., area, child age, feeding, education, and income)

The results for secondary outcome measures are shown in Table 3. Scores for crying knowledge were higher among mothers in the intervention group than those in the control group (61.7 vs. 56.5 points, adjusted difference: 5.11 points, 95% CI: 3.60–6.63, *p* < 0.001). The difference remained significant after adjusting for demographic variables. Similarly, scores for shaking knowledge were higher among the intervention group than the control group (97.4 vs. 92.9 points, adjusted difference: 4.08 points, 95% CI: 2.71–5.45, *p* < 0.001). However, coping behaviors in response to unsoothable crying showed no significant difference between the intervention and control groups.

**Table 3:** Comparison of crying related knowledge and behaviors by intervention and control groups

		Mean (SD)	Crude		Adjusted*	
			Coefficient	95% CI	Coefficient	95% CI
Crying knowledge	Control	56.5 (10.9)	Ref		Ref	
	Intervention	61.7 (11.2)	5.27	3.86–6.68	5.11	3.60–6.63
Shaking knowledge	Control	92.9 (13.0)	Ref		Ref	
	Intervention	97.4 (8.3)	4.12	2.84–5.39	4.08	2.71–5.45
Active coping	Control	10.8 (14.2)	Ref		Ref	
	Intervention	11.4 (14.8)	0.59	−0.73 to 1.91	0.38	−0.96 to 1.72
Self-talk	Control	13.4 (18.9)	Ref		Ref	
	Intervention	12.3 (17.9)	−1.12	−3.00 to 0.76	−1.37	−3.31 to 0.57
		Prevalence (n, %)	Crude		Adjusted*	
			PR	95% CI	PR	95% CI
Walk away	Control	549 (34.6)	Ref		Ref	
	Intervention	399 (37.8)	1.09	0.96–1.24	1.09	0.95–1.25
Sharing crying information	Control	1082 (68.7)	Ref		Ref	
	Intervention	725 (69.4)	1.01	0.92–1.11	1.01	0.91–1.11

*Adjusted for covariates (i.e., area, child age, feeding, education, and income)

### Moderated Intervention Effects

According to the subgroup analysis for shaking, some groups showed a stronger impact of the intervention than the other groups. For example, first child showed lower odds for shaking, but subsequent child showed no intervention effect, and infant with unsoothable crying showed lower odds for shaking, but infant without unsoothable crying showed no intervention effect (Table 4). As for smothering, no differences were observed by subgroup analysis. No significant interaction effect was found between the intervention and education, birth order, and whether the infant cried unsoothably, suggesting the effectiveness of the educational material regardless of maternal education, birth order, or the existence of unsoothable crying.

**Table 4:** Subgroup analysis of the intervention effect by education, birth order, and unsoothable crying

	Odds ratios (intervention group vs. control group) (95%CI)	
Subgroup	Shaking	Smothering
Education	*p* = 0.37	*p* = 0.46
High school or less	0.15 (0.02–1.17)	0.76 (0.07–8.41)
Some college	0.27 (0.03–2.87)	0.94 (0.25–3.50)
College or more	N/A	0.37 (0.08–1.77)
Birth order	*p* = 0.20	*p* = 0.55
First child	**0.07 (0.01–0.56)**	0.82 (0.27–2.49)
Subsequent child	0.46 (0.05–4.42)	0.46 (0.09–2.28)
Unsoothable crying	*p* = 0.72	*p* = 0.98
Yes	**0.09 (0.01–0.78)**	0.75 (0.26–2.17)
No	0.16 (0.02–1.30)	0.72 (0.13–4.10)

**Bold**: *p* < 0.05. *p*: p for interaction term

## Discussion

This study showed that watching an educational video on infant crying and the dangers of shaking and smothering within 1 week after delivery at maternity wards significantly reduced self-reported shaking at 1 month of age by 88%. Further, 42% of smothering behaviors at that age were also reduced, although this change was non-significant. This significant reduction was universal regardless of maternal education, birth order, or having an infant with unsoothable crying.

### Added Evidences to Prevent Shaking and Smothering

The findings of the current study are consistent with those of previous preventive studies on AHT. Dias et al. showed a 51% reduction in AHT in upper New York state using a hospital-based design, i.e., participants were recruited through maternity wards, and were shown an educational video on the dangers of shaking, titled “Portrait of Promise” (Dias et al. 2005). Further, the “Period of PURPLE Crying” was shown to be effective in significantly increasing walk-away behaviors in a randomized controlled trial in Seattle, the United States, and Vancouver, Canada (Barr et al. 2009a, b). This study was replicated in Japan (Fujiwara et al. 2012). Also, the effectiveness of an educational video and pamphlet in real public health settings was confirmed in a previous study using crying and shaking knowledge and walk-away behavior as outcomes (Fujiwara 2015).We add to the literature that educational material to prevent shaking and smothering disseminated at maternity wards during hospitalization after delivery is effective to prevent shaking, but not smothering, behaviors at 1 month of age.

The results of the secondary outcomes may explain the significant protective effect of the educational material. Consistent with previous studies (Barr et al. 2009a, b), we confirmed that those who watched the educational video on infant crying showed higher scores on knowledge of infant crying and shaking. However, we did not find significantly higher scores on coping with infant crying, that is, active coping, self-talk, or walk-away behaviors. In our video, we showed a simulation of the anatomical and dynamic mechanism of shaking using computer graphics and an anatomical doll coupled with commentary from a physical science researcher. This was not featured in previous interventions, such as “Portrait of Promise” which included computer graphics, but not anatomical dolls. The realistic visual simulation depicted in our video and the easy-to-understand commentary on the physics of shaking may have a stronger impact on viewers’ awareness and understanding of the dangers of shaking an infant and may actually reduce shaking behaviors. Thus, rather than focusing on how to manage infant crying, it might be more effective to show parents the danger of shaking their infant by exposing them to a more visual and realistic medium.

Alternatively, the lack of a significant difference in terms of active coping, self-talk, walk away, and sharing crying information may be attributed to the timing of the assessment, that is, we assessed the effectiveness at 1 month of age, which might be too early to assess coping behavior for infant crying (Barr 2012). Nonetheless, we found a significant reduction in self-reported shaking behavior among those who have increased their knowledge of infant crying and shaking by watching the educational video, which suggests that knowledge of crying and shaking, but not active coping behavior, explains the video’s effectiveness to reduce shaking behaviors.

The educational video used in this study would be a useful tool for staff without specific training. Notably, it is freely accessible (available on YouTube), is short in duration (11 mins), and has high fidelity with around 90% of the intervention group remembering that they watched the video.

We note the difficulty in comparing this video with previous educational material “The Period of PURPLE Crying” (Barr et al. 2009a) which requires staff training and comes at a nominal cost of USD3.50 for the Japanese version. Also, as the video in this study was developed only in the Japanese language, translation into other languages, including English, is needed to examine its effectiveness in other countries for international applicability.

### Limitations

Our study has several limitations. First, although we employed cluster randomization, demographic factors differed among those who responded to the questionnaire, possibly because the target sample in intervention may be reluctant to respond to the questionnaire to avoid being suspected for shaking or smothering. Second, the primary outcome of shaking and smothering behaviors was self-reported, and participants were not blinded. Thus the finding might be overestimated due to social desirability. In addition, the interpretation of the terms “shaking” and “smothering” may vary between individuals, although we clearly defined that shaking is “violent shaking due to unsoothable crying” and smothering is “putting your hands or a cushion over the baby’s mouth to stop him/her from crying”, and previous studies in Japan using the same questions reported a similar prevalence of shaking and smothering (Fujiwara et al. 2016a, b; Yamada and Fujiwara 2014). Third, penetration may not be enough, as 9.1% of the intervention group responded that they did not watch the educational video, and contamination might exist, as 14.8% of the control group responded that they watched the educational video, which may have resulted in an underestimation of its effectiveness. Nonetheless, our intention-to-treat analysis of a population-based cluster randomized controlled trial found a strong protective effect for shaking at 1 month of age.

## Conclusions

Screening an educational video on infant crying and the dangers of shaking and smothering within 1 week after delivery at maternity wards reduced the prevalence of self-reported shaking by 88% at 1 month of age. Further studies are needed to replicate the effectiveness of the educational video on the reduction of hospitalized AHT cases.

## Electronic supplementary material

ESM 1 (DOCX 21 kb)
