# Supplementary material for: Effectiveness of an Educational Video in Maternity Wards to Prevent Self-Reported Shaking and Smothering during the First Week of Age: A Cluster Randomized Controlled Trial
Source: Prev Sci. 2020 Jul 22;21(8):1028–36. doi: 10.1007/s11121-020-01145-z (PMC7569084; doi:10.1007/s11121-020-01145-z)
Supplement: Supplementary file 1 — (DOCX 21 kb) [file 11121_2020_1145_MOESM1_ESM.docx]

Journal: Prevention Science

Title: Effectiveness of an Educational Video in Maternity Wards to Prevent Self-reported Serious Infant Abuse in Maternity Wards during the First Week of Age: A Cluster Randomized Controlled Trial

Authors: Takeo Fujiwara^1,2^, PhD, MPH; Aya Isumi^1^, PhD; Makiko Sampei^1,2^, MPH; Yusuke Miyazaki^3^, PhD; Fujiko Yamada^1^, PhD; Hisasi Noma^4^, PhD; Kazuhide Ogita^5^, PhD; Nobuaki Mitsuda^6^, PhD

Affiliations: ^1^ Department of Global Health Promotion, Tokyo Medical and Dental University, Tokyo; ^2^ Department of Social Medicine, National Research Institute for Child Health and Development, Tokyo, Japan; ^3^ Department of Systems and Control Engineering, Tokyo Institute of Technology, Tokyo, Japan; ^4^ Department of Data Science, The Institute of Statistical Mathematics, Tokyo, Japan; ^5^ Department of Obstetrics and Gynecology, Rinku General Medical Center, Osaka, Japan; ^6^ Department of Obstetrics, Osaka Medical Center and Research Institute for Maternal and Child Health, Osaka, Japan

Address correspondence to: Takeo Fujiwara, MD, PhD, MPH, Department of Global Health Promotion, Tokyo Medical and Dental University. [fujiwara.hlth@tmd.ac.jp].

Supplementary 1. Protocol for cluster randomized trial to evaluate the effectiveness of educational material to prevent shaken baby syndrome in obstetric clinic

**1. Purpose**

Shaking and smothering behaviors are known to be triggered by prolonged infant crying. Educational material on infant crying can have a preventive effect when provided appropriately. We aim to evaluate the effectiveness of educational material focusing on feature of infant crying to prevent abusive behaviors such as shaking and smothering at obstetric clinic.

**2. Study Procedure**

**2-1. Participants**

Mothers who delivered their babies in obstetric clinic in Osaka prefecture, Japan, where agreed to participate the study, between October 2014 and January 2015. Based on previous study, prevalence of self-reported shaking can be estimated as 3%, and assuming effect size as 0.5 to 0.7, and power range from 70% to 90%, we estimated the sample size should be approximately 3000. Participants who submit the anonymous response on the questionnaire was considered as agreed to participate the study, which was written in the face sheet of the questionnaire, This opt-out option procedure to obtain informed consent was approved by Ethics Committee in National Center for Child Health and Development.

| Effect size =0.5 |  |  |  |
| --- | --- | --- | --- |
| Power | 70% | 80% | 90% |
| N | 2672 | 3328 | 4368 |
| Effect size =0.6 |  |  |  |
| Power | 70% | 80% | 90% |
| N | 1782 | 2208 | 2882 |
| Effect size=0.7 |  |  |  |
| Power | 70% | 80% | 90% |
| N | 1254 | 1544 | 2006 |

**2-2. Recruitment**

All obstetric clinic in Osaka prefecture were asked to participate the study to show the educational video in maternity wards to mothers after delivery when they were admitted to the hospital or during 1-month health check up.

**2-3. Randomization**

Obstetric clinics agreed to participate in the study were assigned randomly using stratified block randomization. First, we stratified by hospital function (primary or secondary/tertiary hospital), and further stratified by seven region of Osaka prefecture, based on administrative classification (Hokusetsu, Osaka city, Kita-kawachi, Naka-gawachi, Minami-kawachi, Senhoku, and Sennan). Because tertiary hospital covers wider area in Osaka prefecture, for tertiary hospital, “Hokusetsu and Kita-kawachi”, “Naka-gawachi and Minami-gawachi”, and “Senhoku and Sennan” were applied, thus stratified by four regions.

**2-4. Intervention**

The educational video “Baby Doesn’t Stop Crying” was developed by Ministry of Health, Labour, and Welfare in Japan, in collaboration with Drs Takeo Fujiwara, Fujiko Yamada, and Yusuke Miyazaki, based on existing material, the Period of PURPLE Crying by the National Center on Shaken Baby Syndrome (can be viewed on YouTube (in Japanese) at: <https://www.youtube.com/watch?v=T09gzgGUOnY&feature=c4-overview&list=UUVgZUHlkoN51FOwoNMBGjfw>). The video explains patterns of infant crying in healthy infants, including ‘peak crying’, which occurs during the second month and then declines. A computer graphic simulation of the infant brain and an anatomical doll are then used to show the devastating impact that occurs inside the infant skull when a baby is shaken, including the velocity of the skull and brain during shaking. Further, we recommended several ways to soothe infants, such as holding, feeding, swaddling, use of rhythmic stimulation, or taking a break, such as stepping out of the room and away from the crying infant, followed the coping strategies of the Period of PURPLE Crying. The video also emphasized the importance of never shaking or smothering an infant.

In intervention clinic, mothers watch the educational video during their admission after delivery, approximately within 1-week after delivery.

To address ethical concerns, participants in the control group viewed the educational video after submitting the questionnaire at the 1-month health checkup.

**2-5. Evaluation procedure**

Obstetricians or nurses distributed questionnaires to mothers at discharge, and they were asked to return the completed questionnaire to the hospital at the 1-month health checkup.

**3. Eligibility Criteria**

**3-1. Inclusion criteria**

All mothers who deliver babies at obstetric clinics in Osaka which joined this study.

**3-2. Exclusion criteria**

Mothers who delivered still birth and delivered at <22 weeks gestational age.

**4. Outcome Measures and Statistical Methods**

**4-1. Primary outcome measures**

Our primary outcomes were abusive behaviors, that is, shaking and smothering. Shaking behavior was assessed the frequency of shaking behavior in the past month using the following statement: “When your child is crying and making a scene, how many times have you violently shaken your child?”. We used the Japanese term for ‘violently shaking’ instead of ‘shaking’ in the questionnaire because the Japanese term ‘shaking’ can be misinterpreted as ‘rocking’. Similarly, frequency of smothering was assessed with the following question: “How many times have you covered the mouth of your baby with your hands, a cushion, etc., when he/she was crying?”. The respondents selected their answer for each of these questions from the following response items during the past month: “0 times,” “1 or 2 times,” “3–5 times,” “6–10 times,” and “11 or more times”. In our analysis, these responses were dichotomized as 1 for 1 time or more and 0 for 0 times. Further, infant abuse, either shaking or smothering, was also used as an outcome.

**4-2. Secondary outcome measures**

Six secondary outcomes were assessed: crying knowledge, shaking knowledge, sharing information on crying with at least one other family member, walking away, active coping, and self-talk in response to inconsolable crying. Crying and shaking knowledge scales were composed of 6 and 2 questions respectively, asking about knowledge of infant crying properties (e.g., “infant crying increases in the first few weeks of life and reaches a peak in the first 2 or 3 months before getting less.”) and shaking knowledge (e.g. “shaking a baby can be very dangerous and can cause serious injuries”) with 4 Likert type response options (i.e., Strongly Agree, Agree, Disagree, and Strongly Disagree) assigned values of 0–3 respectively, with the correct answer denoted by a higher score. Sharing of information behaviors defined as the per cent of mothers who shared information with crying properties. Walking away behavior was asked by the following question: “During the past month, how many times have you ever put your baby in safe place and walk away when your baby crying inconsolably?” with 5-Likert scale response items of “did not do it”, “once or twice”, “three to five times”, “six to 10 times”, and “11 or more times”. Active coping (e.g. “took the baby for a walk or drive”) and self-talk (e.g. “told yourself the crying would end”) in response to inconsolable crying probed for how mothers responded to unsoothable infant crying with 4 questions on each scale, respectively. Response options were 6-Likert scale, “did not do it”, “once or twice”, “three to five times”, “six to 10 times”, “11 times or more” and “almost every day” during the past month, and scored 0–5 with higher scores indicating higher frequencies. Then, the score for each scale was summed and transformed to a range of 0–100 with higher scores indicating better knowledge or improved behaviors. Sharing crying information and walk-away behaviors were dichotomized, whether the behaviors occurred 0 times or 1 or more times.

**4-3. Statistical Methods**

We employed intention-to-treat analysis. For continuous measures, the mean difference between participants in the intervention group and the control was estimated using a multilevel regression model with a restricted maximum likelihood method to take into account clustering of the hospital. For dichotomized outcomes, we estimated the odds ratio (OR) using a multilevel logistic regression model. Following the previous randomized controlled trials, we used tests of statistical interaction for our primary outcomes to examine subgroups based on education (high school or less vs. some college or more), parity (first child vs. subsequent child), and whether the infant cried inconsolably. Stata SE version 14 was used for analysis. All tests were two-sided with a significance level of p<0.05.
